# On the importance of Task 1 and error performance measures in PRP dual-task studies

**DOI:** 10.3389/fpsyg.2015.00403

**Published:** 2015-04-07

**Authors:** Tilo Strobach, Anja Schütz, Torsten Schubert

**Affiliations:** ^1^Department of Psychology, Humboldt University Berlin, Berlin, Germany; ^2^Department of Psychology, Medical School Hamburg, Hamburg, Germany

**Keywords:** PRP, dual tasks, capacity limitation, central bottleneck theory, reaction times, error data

## Abstract

The psychological refractory period (PRP) paradigm is a dominant research tool in the literature on dual-task performance. In this paradigm a first and second component task (i.e., Task 1 and Task 2) are presented with variable stimulus onset asynchronies (SOAs) and priority to perform Task 1. The main indicator of dual-task impairment in PRP situations is an increasing Task 2-RT with decreasing SOAs. This impairment is typically explained with some task components being processed strictly sequentially in the context of the prominent central bottleneck theory. This assumption could implicitly suggest that processes of Task 1 are unaffected by Task 2 and bottleneck processing, i.e., decreasing SOAs do not increase reaction times (RTs) and error rates of the first task. The aim of the present review is to assess whether PRP dual-task studies included both RT and error data presentations and statistical analyses and whether studies including both data types (i.e., RTs and error rates) show data consistent with this assumption (i.e., decreasing SOAs and unaffected RTs and/or error rates in Task 1). This review demonstrates that, in contrast to RT presentations and analyses, error data is underrepresented in a substantial number of studies. Furthermore, a substantial number of studies with RT and error data showed a statistically significant impairment of Task 1 performance with decreasing SOA. Thus, these studies produced data that is not primarily consistent with the strong assumption that processes of Task 1 are unaffected by Task 2 and bottleneck processing in the context of PRP dual-task situations; this calls for a more careful report and analysis of Task 1 performance in PRP studies and for a more careful consideration of theories proposing additions to the bottleneck assumption, which are sufficiently general to explain Task 1 and Task 2 effects.

## Introduction

When people execute two simultaneous or systematically delayed distinct tasks under dual-task conditions, performance in these tasks is often impaired (e.g., Kahneman, 1973; Wickens, 1980; Pashler, 2000, and many more). In the context of well-controllable behavioral dual-task situations, these impairments are demonstrated by an increase in reaction times (RTs) and/or error rates under dual-task in contrast to single-task conditions (the isolated task execution), referred to as “dual-task costs.”

**FIGURE 1:**
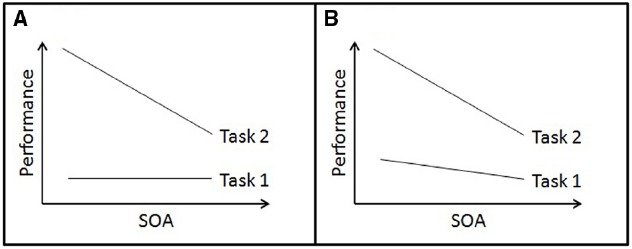
**Illustration of performance patterns in the context of dual tasks of the Psychological Refractory Period type (note, that an increase at the y axis represents performance impairment and an increase at the x axis represents an increase in stimulus onset asynchrony, SOA).** Panel **(A)**: Task 1 and Task 2 performance according to the central bottleneck model. Panel **(B)**: Task 1 and Task 2 performance according to less strict sequential interpretations of bottleneck models and impaired performance with decreasing SOA in both tasks.

One of the most prominent dual-task situations is of the psychological refractory period (PRP) type (Telford, 1931; Vince, 1949; Welford, 1952; Pashler, 1984, 1994; Pashler and Johnston, 1989, 1998; Osman and Moore, 1993; Schubert, 1999; Schubert et al., 2008). In this dual-task situation, two component tasks are presented in close succession with various time intervals between the onsets of a first and second task stimulus (i.e., variable stimulus onset asynchronies, SOAs) and participants are given fixed-priority instructions on the execution of the first task (Task 1). As illustrated in Figure 1A, the performance of the second task (Task 2) typically decreases (e.g., RTs increase) with decreasing SOA and increasing task overlap. This performance decrease indicates dual-task costs in the context of PRP dual tasks (i.e., the PRP effect).

To explain the PRP effect, the prominent *central bottleneck theory* suggests that the mental operation associated with the selection of which response to execute can never be made for two tasks simultaneously. Instead, this model assumes that response selection requires a single mechanism to be dedicated to it for some period of time. Thus, there is a strict sequential response selection in two tasks of a dual-task situation due to a structural and unavoidable processing bottleneck. In this strict interpretation of the central bottleneck model, the sequential processing at the central bottleneck leads to processing impairments (i.e., processing delays or errors) in the second component tasks of a PRP situation. This processing impairment increases with decreasing SOA (Pashler, 1994), leading to the PRP effect in Task 2.

Accordingly, the literature on PRP/central bottleneck theory typically introduces and entails no effect of SOA on Task 1 performance (Figure 1A; e.g., Pashler, 1984, 1994; Allen et al., 1998; Ruthruff and Pashler, 2001; Kunde et al., 2007, and many more). This lacking effect is a consequence of the assumption of a strict sequential processing of response selections, i.e., (1) an engagement of a central processing mechanism of 100 and 0% to Task 1 and Task 2, respectively, before (2) an engagement of this mechanism of 0 and 100% to Task 1 and Task 2, respectively. Task 1 and Task 2 performance analyses should thus be treated in the context of PRP situations and the central bottleneck theory in order to test these assumptions. Furthermore, these analyses should focus on all available data types. In the context of most behavioral experiments, these analyses combine analyses on RTs as well as error rate data.^1^

The dual-task literature, however, seems to treat (1) Task 1 performance and (2) error rates with less emphasis and caution. One of the few examples that systematically relates dual-task performance in form of error rates to RTs occurs in a series of studies combining ideomotor compatibility tasks (i.e., component tasks in which stimulus information shares response information; e.g., if an auditory stimulus is “A” or “B,” say “A” and “B,” respectively) in PRP situations (Lien et al., 2002; Greenwald, 2003). Dual-task performance showed an effect of SOA on error rates regardless of the type of instruction (Shin et al., 2007). These rates increased from long to short SOAs under conditions of a speed-instruction (i.e., “Perform as fast as possible”) as well as under a speed-and-accuracy instruction (i.e., “Perform as fast and as accurately as possible”) in both Task 1 and Task 2. The authors interpreted these findings (in combination with RT data) as evidence for bottleneck processing when combining ideomotor compatible tasks. Alongside this example, there are only very few approaches in PRP literature and literature with strict interpretations of the central bottleneck theory that systematically apply and interpret error data with a focus on Task 1 performance for modeling dual-task processing. This is surprising considering that alternative theoretical approaches within the context of the central bottleneck theory (see Discussion) and outside this context (e.g., resource theories, Kantowitz, 1978; Logan and Gordon, 2001; Navon and Miller, 2002; Wickens, 2002; Tombu and Jolicœur, 2003) particularly provide systematic interpretations of Task 1’s error rates as well as RTs. The consideration of Task 1’s data is essential to produce a comprehensive picture of dual-task processing in the context of these theories.

As a consequence of the impression of treating Task 1 performance with less emphasis and caution, the aim of the present study is to systematically review PRP literature with a focus on (1) the report policy of Task 1 performance data as well as (2) the actual performance in this task. The first aim generally specifies the policy to report error data compared to RT data of Task 1. Importantly, we specify this policy with a focus on presenting RT and error data (e.g., in form of figures and/or tables), as well as reporting statistics on both performance measures (e.g., in form of analysis of variance). While the central bottleneck theory makes explicit assumptions on RTs, error rates are often underrepresented in the context of this theory. Therefore, we speculate that, due to this underrepresentation, the number of PRP studies including reported error data (i.e., in form of data presentations and statistical analyses) is lower than the number including RT data (despite a general request of no selective data report in empirical studies, Boot et al., 2011).

We review whether Task 1 performance is independent of SOA using the perspective of the second aim: that is, RTs and error rates are constant across SOAs. On the other hand, the following data patterns in Task 1 are not conceivable in the context of this theory: (1) RTs are constant and error rates increase with decreasing SOA, (2) error rates are constant and RTs increase with decreasing SOA, (3) error rates and RTs increase with decreasing SOA (Figure 1B). These latter data patterns are consistent with the assumption that bottleneck processing is potentially less sequential than theorized in a strict interpretation of the central bottleneck theory, but they call for additions to this assumption (as indicated in the Discussion).

We aim to investigate the proportion of PRP studies that are consistent with one of these data patterns in the present review. In detail, we analyzed the number of PRP experiments showing a statistically significant main effect of SOA (typically in ANOVAs) on Task 1’s RTs and/or error rates, in combination with data patterns demonstrating impaired performance with decreasing SOA (i.e., increased RTs and/or error rates with shorter SOA).^2^ PRP dual-task situations are rigorous tests of these patterns in Task 1, since participants are explicitly instructed to prioritize this task. This priority on Task 1 should make this task less vulnerable for performance modulations due to the timing (i.e., SOA) of the following Task 2 and should reduce the likelihood of impaired Task 1 performance with decreasing SOAs. Note that we exclusively review SOA main effects on RTs and/or error rates because we focus on robust effects. This focus on robust effects parallels the robust emergence of PRP effects in Task 2 and their clear demonstration via SOA main effects. Furthermore, our focus on SOA main effects allows us to combine analyses across different studies in which SOA modulation is often combined with one or a set of alternative factors (e.g., modulations of stimulus characteristics, stimulus-response relations, etc.). Generally, this type of review should help advance the dual-task literature by specifying the dual-task processing architecture of PRP dual tasks. Furthermore, this review investigates the benefits of using error rates and/or Task 1 performance as a data source to improve our insight into dual-task processing and its theory.

## Methods

We searched papers via the abstracting and indexing database PsycINFO devoted to peer-reviewed literature in the behavioral sciences and mental health on May 17th 2013. The search term was “PRP.” This search resulted in a total number of 291 entries from which we excluded reviews, clinical papers, dissertation abstracts, book chapters, modeling studies, and non-English entries. This exclusion procedure left a selection of 133 studies. In total, these studies comprised 306 experiments.

## Results

In the Results section, we first focus on the amount of papers presenting Task 1’s RT data vs. error rates in form of figures and/or tables. Secondly, we report the amount of studies that perform inference-statistical analyses (e.g., analysis of variance) on RTs and/or error rates of this task. Thirdly, we review PRP studies with a particular focus on the impact of decreasing SOA on decreasing performance (i.e., increasing RTs and error rates) in Task 1.

The number and percent of experiments (out of the total of 306 experiments of 133 studies) presenting RT or error data in form of (1) figures, (2) tables, (3) figures and tables, as well as (4) figures and/or tables for Task 1, is presented in Table 1. Apart from what PRP studies include and is presented in this table, the reversed perspective on this table is remarkable: 181 (59.2%) of all experiments [and 97 (45.3%) of all experiments with presentation of Task 1 RT data] presented no error data. In contrast, only 92 (30.1%) of all experiments [and 8 (6.4%) of all experiments with presentation of error data] presented no RT data. Thus, this review demonstrates the underrepresentation of error data presentation in contrast to presenting RTs of Task 1 in PRP dual-task studies.

**TABLE 1:** **Number and percent of experiments presenting RTs and error rates in figures, tables, figures and tables, as well as figures or tables**.

	Figures	Tables	Figures and tables	Figures or tables
RTs	177 (57.8%)	72 (23.5%)	38 (12.4%)	214 (69.9%)
Error rates	19 (6.2%)	108 (35.3%)	2 (0.7%)	125 (40.8%)

Total number of experiments is 306.

The number and percent of experiments (out of a total of 306 experiments of 133 studies) with statistical analyses of RT or error data is presented in Table 2. From our perspective, the most crucial fact of this table is that only 48.7% of the experiments provided statistical analyses of their RTs and error rates. On the other side, this table shows that there is no complete presentation of statistical analyses for 51.3% of the experiments. Thus, this review demonstrates that many PRP dual-task studies allow no conclusive conclusions about Task 1 performance and no test of the implicit assumption that this task’s performance is independent of SOA.

**TABLE 2:** **Number and percent of experiments (total number experiments is 306) with statistical analyses of (1) RTs, (2) error data, (3) RTs and error data, as well as (4) RTs and/or error data in Task 1**.

	RTs	Errors	RTs and errors	RTs and/ or errors
Task 1 statistics	227 (74.2%)	166 (54.2%)	149 (48.7%)	234 (76.5%)

While focusing on the third issue, we exclusively analyzed the selection of studies (combining 149 experiments, see Appendix) that provided statistical analyses of their Task 1’s RTs and error rates (Table 3). This focus shows that a remarkable number of PRP dual-task studies produced data that are not consistent with the assumption of a strict sequential bottleneck processing but calls for additions to this bottleneck assumption (Figure 1B): Task 1 performance was not independent of SOA in most of these studies and showed impaired performance with decreasing SOA (67.1%).

**TABLE 3:** **Number and percent of experiments (total number experiments is 149) with statistical analyses of RTs and error data (see Table 2) as well as an effect of SOA on Task 1 performance (i.e., decreasing SOA and increasing RTs/error rates)**.

	RTs	Errors	RTs and errors	RTs and/ or errors
Task 1 SOA effect	55 (36.9%)	73 (49.0%)	28 (18.8%)	100 (67.1%)

## Discussion

Our review demonstrates that a lot of studies do not present all data that is required to analyze and model dual-task processing in the context of the central bottleneck theory in the case of PRP dual-task experiments. First, while a reasonable amount of studies presented RT data of both component tasks (69.3%), this amount is drastically reduced for error data: only 40.8% of the PRP studies presented this data type in tables and/or figures. This rather low amount of studies including error data presentations demonstrates that there is no obtainable conclusive interpretation of PRP dual-task performance in many studies. Thus, these studies do not allow to completely model dual-task processing in the context of the central bottleneck theory. Furthermore, we found a rather low number of studies that analyzed this data statistically and allowed conclusive conclusions about in this context. At this point, it is however fair to admit that not all studies that were identified based on our literature search on “PRP” pursued on investigating the central bottleneck theory; the PRP paradigm can be used manifold (e.g., to simply induce capacity constraints to Task 2 processing). In such cases, reports, analyses, and interpretations should mainly focus on relevant aspects (e.g., primarily data of Task 2).

If we included studies with statistical reports on error data and RTs, a substantial number of experiments demonstrated that, with decreasing SOA, there are increases of error rates, increases of RTs, or both in Task 1. In fact, 67.1% of the included experiments demonstrated one of these patterns. We assume that this number could be even higher because (1) experiments with SOA null effects in Task 1 may merely lack statistical power to reach the significance threshold of a SOA main effect and/or (2) studies with no impaired performance with decreasing SOA also include studies showing an opposite pattern: performance impairments with increasing SOA (see text Footnote 2). This pattern may, however, demonstrate the impact of a response grouping strategy (e.g., Borger, 1963; Schubert, 1996; Miller and Ulrich, 2008). This strategy may mask a data pattern of an impaired performance with decreasing SOA and thus may obscure the number of studies including this pattern. Moreover, for reasons of comparability, we exclusively focused on SOA main effects and neglected combinations of these effects with alternative experimental factors (i.e., interactions). The extension of the focus to interactions could potentially increase the number of experiments with performance impairments of Task 1 with decreasing SOA (particularly when the SOA main effect is non-significant).

Nevertheless, there are a number of theories that explicitly consider dual-task costs in Task 1 (in the PRP context: performance impairments at short in contrast to long SOAs). First, *capacity-sharing theories* assume that two response selections can be processed in parallel, but that sharing the same limited resource(s) causes dual-task costs (e.g., Herman and Kantowitz, 1970; Kantowitz and Knight, 1974, 1976; Kantowitz, 1978; Wickens, 2002; Tombu and Jolicœur, 2003), because there are fewer resources for each individual task and performance is thus impaired. Participants strategically prioritized one task over another following instructions and/or changes in the relationship of difficulty between the combined tasks, which is consistent with this perspective (e.g., Norman and Bobrow, 1975; Navon and Gopher, 1979; Gopher et al., 1982). Recent representatives of capacity-sharing theories (e.g., Logan and Gordon, 2001; Navon and Miller, 2002; Tombu and Jolicœur, 2003) assume that sequential processing, as anticipated in the central bottleneck theory, may be a strategic product of flexible scheduling of limited resources. For example, participants may not have followed the instruction of PRP dual tasks strictly scheduling engagement 100% to Task 1 and 0% to Task 2 adequately. Task 1 effects can also be explained by the assumption of a strategic task scheduling with a flexible bottleneck localization during the task processing and resource sharing at the level of executive control processes (Meyer and Kieras, 1997).

Second, dual-task costs in Task 1 were also explained in terms of between-task *crosstalk* (e.g., Hommel, 1998; Logan and Schulkind, 2000; Logan and Gordon, 2001; Lien and Proctor, 2002; Navon and Miller, 2002; Schubert et al., 2008). For instance, performance decreases when two tasks require the simultaneous execution of incompatible (e.g., left vs. right) in contrast to compatible (e.g., left vs. left) spatial responses. This crosstalk assumption is generally consistent with the assumption of capacity-sharing theories, since both enable information transfer between component tasks under dual-task conditions. Thus, if some of the studies demonstrating Task 1 performance impairment with decreasing SOA showed this impairment because of crosstalk, this finding is consistent with sharing common resources between tasks. The difference between crosstalk approaches and capacity-sharing theories is however that the former depend on what content of information is processed while dual-task costs depend on what sort of operation is to be carried out is interpreted in the latter capacity-sharing context. Interestingly, the approaches of Hommel (1998), Lien and Proctor (2002), as well as Schubert et al. (2008) propose a distinction of different sub-processes of the response selection mechanisms (e.g., response activation and initiation), which to different degrees are subjected to cross-task and to sequential processing between tasks. This allows explaining a range of the reported Task 1 effects, with elaborated bottleneck models.

Third, the processing bottleneck (in form of a shared capacity limitation or a structural bottleneck as in the central bottleneck theory) requires the coordination of two task processing streams. For instance, these task coordination processes are related to the efficient preparation of Task 1 information (de Jong, 1995), scheduling of response selections, as well as switches between them (Umiltà et al., 1992; Schubert, 1996, 2008; Lien et al., 2003; Band and van Nes, 2006; Sigman and Dehaene, 2006; Szameitat et al., 2006; Liepelt et al., 2011; Strobach et al., 2012b, 2014). We assume that the latter set of mechanisms (i.e., task coordination processes) particularly affects Task 1 processing under conditions of uncertainty of task order processing (e.g., Arnell and Duncan, 2002, Experiment 1; de Jong, 1995; Tombu and Jolicœur, 2002; Luria and Meiran, 2003, 2005, 2006; Sigman and Dehaene, 2006; Schubert, 2008; Leonhard and Ulrich, 2011; Strobach et al., 2012a; Töllner et al., 2012) because the decision on the order of bottleneck access is typically located before bottleneck processing in Task 1 (Umiltà et al., 1992; Schubert, 1996; Hendrich et al., 2012). The number of studies with task order uncertainty is, however, rather low in comparison to the entire set of analyzed PRP studies and thus should not obscure our general conclusion: a substantial number of experiments in the context of PRP dual-task experiments demonstrate decreasing Task 1 performance with decreasing SOA, which is not consistent with the assumption that processes of Task 1 are unaffected of Task 2 and bottleneck processing in the context of PRP dual-task situations. Actually, this calls for a more careful consideration of theories proposing additions to the bottleneck assumption, which are sufficiently general to explain Task 1 and Task 2 effects.

### Conflict of Interest Statement

The authors declare that the research was conducted in the absence of any commercial or financial relationships that could be construed as a potential conflict of interest.

## Footnotes

1 Here, erroneous dual-task performance is defined as giving an incorrect response or omitting a response in a particular component task and with separate error rate analyses on Task 1 and Task 2. This perspective does not consider studies in which any error that occurred during dual-task performance (irrespective of the component task) meant that the dual task as a whole was not performed correctly and thus error analysis was conducted for both tasks in combination (e.g., Logan and Schulkind, 2000; Strobach et al., 2013; Zwosta et al., 2013). We take this perspective, because the combined error analysis does not allow elaborated and independent conclusions for Task 1 and Task 2 performance.

2 We were not interested in the reverse phenomenon of impaired performance with increasing SOA (i.e., decreased RTs/error rates at short in contrast to long SOAs) which is typically discussed in the context of grouping of two responses/a tendency to withhold the execution of a first response until the execution of a second response (Borger, 1963; Miller and Ulrich, 2008).
